# Dichlorido{1-[(2-hy­droxy­eth­yl)imino­meth­yl]-2-naphtho­lato}pyridine­iron(III) pyridine monosolvate

**DOI:** 10.1107/S1600536810046623

**Published:** 2010-11-17

**Authors:** Shizheng Liu, Jie Yang, Lei Lv, Dacheng Li

**Affiliations:** a School of Chemistry and Chemical Engineering, Liaocheng University, Shandong 252059, People’s Republic of China

## Abstract

In the title complex, [Fe(C_13_H_12_NO_2_)Cl_2_(C_5_H_5_N)]·C_5_H_5_N, the iron(III) atom is six-coordinated by the N and O atoms from the Schiff base ligand, the N atom from a pyridine mol­ecule and two chloride anions in a distorted octa­hedral geometry. The crystal packing is stabilized by inter­molecular O—H⋯N hydrogen bonds and C—H⋯π inter­actions.

## Related literature

For the synthesis and applications of magnetic complexes, see: Oshio *et al.* (2004 ▶); Aromí & Brechin (2006 ▶). For related structures, see: Goodwin *et al.* (2000 ▶); Qian *et al.* (2008 ▶); Hoshino *et al.* (2009 ▶).
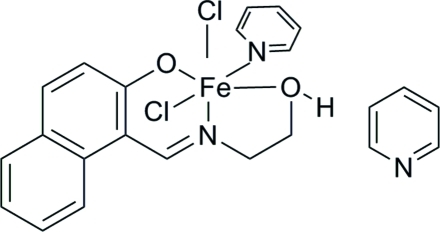


## Experimental

### 

#### Crystal data

[Fe(C_13_H_12_NO_2_)Cl_2_(C_5_H_5_N)]·C_5_H_5_N
*M* *_r_* = 499.19
Monoclinic, 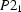

*a* = 7.8590 (6) Å
*b* = 10.0153 (11) Å
*c* = 14.4884 (16) Å
β = 90.123 (1)°
*V* = 1140.4 (2) Å^3^
*Z* = 2
Mo *K*α radiation
μ = 0.92 mm^−1^
*T* = 298 K
0.48 × 0.44 × 0.37 mm

#### Data collection

Bruker SMART 1000 CCD diffractometer
Absorption correction: multi-scan (*SADABS*; Siemens, 1996 ▶) *T*_min_ = 0.666, *T*_max_ = 0.727
5726 measured reflections
3395 independent reflections
3143 reflections with *I* > 2σ(*I*)
*R*_int_ = 0.021

#### Refinement

*R*[*F* ^2^ > 2σ(*F* ^2^)] = 0.032
*wR*(*F* ^2^) = 0.085
*S* = 1.00
3395 reflections
281 parameters
1 restraint
H-atom parameters constrained
Δρ_max_ = 0.25 e Å^−3^
Δρ_min_ = −0.19 e Å^−3^
Absolute structure: Flack (1983 ▶), 1265 Friedel pairs
Flack parameter: 0.02 (2)

### 

Data collection: *SMART* (Siemens, 1996 ▶); cell refinement: *SAINT* (Siemens, 1996 ▶); data reduction: *SAINT*; program(s) used to solve structure: *SHELXS97* (Sheldrick, 2008 ▶); program(s) used to refine structure: *SHELXL97* (Sheldrick, 2008 ▶); molecular graphics: *SHELXTL* (Sheldrick, 2008 ▶); software used to prepare material for publication: *SHELXTL*.

## Supplementary Material

Crystal structure: contains datablocks I, global. DOI: 10.1107/S1600536810046623/rz2519sup1.cif

Structure factors: contains datablocks I. DOI: 10.1107/S1600536810046623/rz2519Isup2.hkl

Additional supplementary materials:  crystallographic information; 3D view; checkCIF report

## Figures and Tables

**Table 1:** Hydrogen-bond geometry (Å, °) *Cg* is the centroid of the C4–C9 ring.

*D*—H⋯*A*	*D*—H	H⋯*A*	*D*⋯*A*	*D*—H⋯*A*
O1—H1⋯N3	0.82	1.81	2.617 (4)	168
C12—H12⋯*Cg*^i^	0.93	2.71	3.594 (5)	159
C16—H16⋯*Cg*^ii^	0.93	2.78	3.653 (6)	157

Symmetry codes: (i) 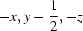; (ii) 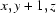.

**Fig. 1.**
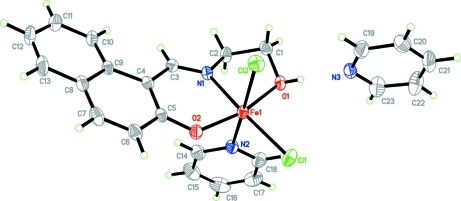
The molecular structure of the title compound, with atom labels and 30% probability displacement ellipsoids.

## supplementary crystallographic information

### Comment

The considerable current interest in magnetic complexes arises from their relevance in diverse fields ranging from bioinorganic chemistry to molecular magnetic materials (Oshio *et al.*, 2004; Aromí *et al.*, 2006). We report here the synthesis and crystal structure of the title compound.

In the title complex (Fig. 1), the tridentate Schiff base ligand is derived from the condensation of 2-hydroxy-1-naphthaldehyde and ethanolamine. The iron(III) metal centre is six-coordinate by the N and O atoms from the Schiff base ligand, the N atom from a pyridine molecule and two chloride anions in a distorted octahedral geometry. The Fe–O(alkoxo) bond length [Fe1–O1 = 2.105 (2) Å] is longer than the Fe–O(phenoxo) distance [Fe1–O2 = 1.897 (2) Å], which lies well within the range of values reported in the literature (Goodwin *et al.*, 2000; Qian *et al.*, 2008; Hoshino *et al.*, 2009). In the crystal structure, complex molecules and pyridine solvent molecules are linked into a three-dimensional network by O—H···N hydrogen bonds and C—H···π interactions (Table 1).

### Experimental

Ethanolamine (1 mmol, 61.08 mg) was dissolved in methanol (10 ml) and added dropwise to a methanol solution of 2-hydroxy-1-naphthaldehyde (1 mmol, 172.19 mg). The mixture was then stirred at 323 K for 2 h. Subsequently, an MeCN/Py solution "(3:1 *v*/*v*, 4 ml) of FeCl_3_.6H_2_O(1 mmol, 270.29 mg) was added dropwise and stirred for another 7 h. The resulting black solution was filtrated and was allowed to stand at room temperature for about one week, whereupon block crystals suitable for X-ray diffraction analysis were obtained.

### Refinement

All H atoms were placed in geometrically idealized positions and treated as riding on their parent atoms, with C—H = 0.93–0.97 Å, O—H = 0.82 Å, and with *U*_iso_(H) = 1.2 *U*_eq_(C) or 1.5 *U*_eq_(O).

### Crystal data

**Table:**

[Fe(C_13_H_12_NO_2_)Cl_2_(C_5_H_5_N)]·C_5_H_5_N	*F*(000) = 514
*M**_r_* = 499.19	*D*_x_ = 1.454 Mg m^−^^3^
Monoclinic, *P*2_1_	Mo *K*α radiation, λ = 0.71073 Å
Hall symbol: P 2yb	Cell parameters from 3504 reflections
*a* = 7.8590 (6) Å	θ = 2.5–28.0°
*b* = 10.0153 (11) Å	µ = 0.92 mm^−^^1^
*c* = 14.4884 (16) Å	*T* = 298 K
β = 90.123 (1)°	Block, black
*V* = 1140.4 (2) Å^3^	0.48 × 0.44 × 0.37 mm
*Z* = 2	

### Data collection

**Table:**

Bruker SMART 1000 CCD diffractometer	3395 independent reflections
Radiation source: fine-focus sealed tube	3143 reflections with *I* > 2σ(*I*)
graphite	*R*_int_ = 0.021
phi and ω scans	θ_max_ = 25.0°, θ_min_ = 1.4°
Absorption correction: multi-scan (*SADABS*; Siemens, 1996)	*h* = −8→9
*T*_min_ = 0.666, *T*_max_ = 0.727	*k* = −11→8
5726 measured reflections	*l* = −17→17

### Refinement

**Table:**

Refinement on *F*^2^	Secondary atom site location: difference Fourier map
Least-squares matrix: full	Hydrogen site location: inferred from neighbouring sites
*R*[*F*^2^ > 2σ(*F*^2^)] = 0.032	H-atom parameters constrained
*wR*(*F*^2^) = 0.085	*w* = 1/[σ^2^(*F*_o_^2^) + (0.0476*P*)^2^ + 0.5289*P*] where *P* = (*F*_o_^2^ + 2*F*_c_^2^)/3
*S* = 1.00	(Δ/σ)_max_ = 0.001
3395 reflections	Δρ_max_ = 0.25 e Å^−^^3^
281 parameters	Δρ_min_ = −0.19 e Å^−^^3^
1 restraint	Absolute structure: Flack (1983), 1265 Friedel pairs
Primary atom site location: structure-invariant direct methods	Flack parameter: 0.02 (2)

### Fractional atomic coordinates and isotropic or equivalent isotropic displacement parameters (Å^2^)

**Table:**

	*x*	*y*	*z*	*U*_iso_*/*U*_eq_	
Fe1	0.54656 (5)	0.51232 (5)	0.28032 (3)	0.03179 (13)	
Cl1	0.81839 (12)	0.59516 (12)	0.27394 (7)	0.0505 (3)	
Cl2	0.61339 (13)	0.29636 (11)	0.33678 (7)	0.0486 (3)	
N1	0.2862 (3)	0.4752 (3)	0.30300 (17)	0.0321 (7)	
N2	0.4541 (4)	0.7190 (4)	0.2447 (2)	0.0386 (7)	
N3	0.7698 (4)	0.5286 (4)	0.52783 (19)	0.0458 (8)	
O1	0.5126 (3)	0.5757 (3)	0.41754 (15)	0.0374 (6)	
H1	0.5968	0.5524	0.4469	0.056*	
O2	0.5074 (3)	0.4537 (3)	0.15755 (16)	0.0386 (6)	
C1	0.3621 (4)	0.5234 (5)	0.4608 (2)	0.0434 (9)	
H1A	0.3330	0.5757	0.5149	0.052*	
H1B	0.3798	0.4315	0.4796	0.052*	
C2	0.2222 (4)	0.5320 (5)	0.3892 (2)	0.0409 (10)	
H2A	0.1231	0.4827	0.4098	0.049*	
H2B	0.1894	0.6244	0.3798	0.049*	
C3	0.1855 (4)	0.4104 (4)	0.2498 (2)	0.0331 (8)	
H3	0.0734	0.4006	0.2690	0.040*	
C4	0.2316 (4)	0.3513 (4)	0.1627 (2)	0.0309 (8)	
C5	0.3927 (4)	0.3731 (4)	0.1223 (2)	0.0334 (8)	
C6	0.4347 (5)	0.3075 (5)	0.0381 (2)	0.0440 (10)	
H6	0.5403	0.3227	0.0113	0.053*	
C7	0.3239 (5)	0.2239 (5)	−0.0032 (2)	0.0505 (11)	
H7	0.3564	0.1808	−0.0572	0.061*	
C8	0.1613 (5)	0.1997 (4)	0.0327 (2)	0.0419 (10)	
C9	0.1090 (5)	0.2669 (4)	0.1152 (2)	0.0330 (8)	
C10	−0.0580 (5)	0.2445 (4)	0.1469 (3)	0.0431 (9)	
H10	−0.0968	0.2911	0.1982	0.052*	
C11	−0.1640 (6)	0.1561 (5)	0.1041 (3)	0.0566 (12)	
H11	−0.2733	0.1426	0.1268	0.068*	
C12	−0.1089 (6)	0.0855 (6)	0.0261 (3)	0.0632 (14)	
H12	−0.1796	0.0221	−0.0011	0.076*	
C13	0.0459 (6)	0.1095 (5)	−0.0094 (3)	0.0576 (12)	
H13	0.0783	0.0656	−0.0631	0.069*	
C14	0.3280 (5)	0.7367 (5)	0.1839 (3)	0.0496 (10)	
H14	0.2786	0.6622	0.1565	0.060*	
C15	0.2690 (7)	0.8615 (6)	0.1604 (3)	0.0659 (14)	
H15	0.1814	0.8708	0.1177	0.079*	
C16	0.3409 (7)	0.9727 (5)	0.2009 (4)	0.0731 (16)	
H16	0.3043	1.0580	0.1851	0.088*	
C17	0.4661 (7)	0.9548 (5)	0.2644 (4)	0.0635 (13)	
H17	0.5147	1.0279	0.2940	0.076*	
C18	0.5204 (5)	0.8271 (5)	0.2843 (3)	0.0509 (10)	
H18	0.6071	0.8160	0.3274	0.061*	
C19	0.8321 (6)	0.4104 (5)	0.5477 (3)	0.0588 (13)	
H19	0.7821	0.3355	0.5211	0.071*	
C20	0.9701 (7)	0.3926 (6)	0.6070 (4)	0.0650 (14)	
H20	1.0113	0.3077	0.6200	0.078*	
C21	1.0421 (5)	0.5014 (8)	0.6448 (3)	0.0656 (14)	
H21	1.1334	0.4929	0.6852	0.079*	
C22	0.9803 (7)	0.6227 (7)	0.6233 (4)	0.0805 (19)	
H22	1.0306	0.6991	0.6476	0.097*	
C23	0.8438 (7)	0.6334 (6)	0.5658 (4)	0.0663 (14)	
H23	0.8009	0.7178	0.5527	0.080*	

### Atomic displacement parameters (Å^2^)

**Table:**

	*U*^11^	*U*^22^	*U*^33^	*U*^12^	*U*^13^	*U*^23^
Fe1	0.0295 (2)	0.0306 (3)	0.0353 (2)	0.0020 (2)	−0.00537 (16)	−0.0014 (2)
Cl1	0.0305 (4)	0.0524 (7)	0.0685 (6)	−0.0014 (4)	−0.0044 (4)	−0.0056 (5)
Cl2	0.0574 (6)	0.0332 (5)	0.0551 (6)	0.0102 (5)	−0.0071 (4)	0.0020 (4)
N1	0.0296 (14)	0.038 (2)	0.0290 (13)	0.0046 (12)	−0.0029 (11)	−0.0044 (12)
N2	0.0401 (17)	0.034 (2)	0.0420 (16)	0.0029 (15)	−0.0066 (13)	0.0025 (15)
N3	0.0476 (17)	0.046 (2)	0.0432 (16)	−0.0030 (18)	−0.0118 (13)	0.0028 (18)
O1	0.0377 (13)	0.0390 (16)	0.0354 (12)	0.0012 (11)	−0.0105 (10)	−0.0038 (11)
O2	0.0354 (13)	0.0449 (17)	0.0355 (12)	−0.0024 (11)	−0.0012 (10)	−0.0040 (11)
C1	0.0451 (18)	0.053 (3)	0.0323 (16)	0.001 (2)	0.0004 (13)	−0.005 (2)
C2	0.0336 (17)	0.051 (3)	0.0381 (17)	0.0047 (18)	−0.0013 (13)	−0.0170 (19)
C3	0.0307 (17)	0.032 (2)	0.0366 (18)	0.0043 (16)	−0.0019 (14)	0.0004 (16)
C4	0.0349 (18)	0.028 (2)	0.0294 (16)	0.0088 (15)	−0.0046 (14)	0.0009 (14)
C5	0.0358 (18)	0.033 (2)	0.0313 (17)	0.0065 (16)	−0.0076 (14)	0.0028 (15)
C6	0.0405 (19)	0.060 (3)	0.0319 (18)	0.006 (2)	0.0015 (15)	−0.0043 (19)
C7	0.060 (2)	0.060 (3)	0.0319 (19)	0.011 (2)	−0.0023 (17)	−0.014 (2)
C8	0.050 (2)	0.042 (3)	0.0339 (18)	0.002 (2)	−0.0114 (16)	−0.0019 (17)
C9	0.0387 (18)	0.029 (2)	0.0310 (16)	0.0043 (15)	−0.0086 (14)	−0.0003 (14)
C10	0.040 (2)	0.047 (3)	0.042 (2)	−0.0033 (18)	−0.0111 (16)	−0.0038 (18)
C11	0.049 (2)	0.066 (3)	0.056 (2)	−0.011 (2)	−0.016 (2)	0.001 (2)
C12	0.071 (3)	0.064 (3)	0.055 (3)	−0.020 (3)	−0.026 (2)	−0.010 (2)
C13	0.074 (3)	0.054 (3)	0.044 (2)	−0.002 (2)	−0.019 (2)	−0.018 (2)
C14	0.053 (2)	0.046 (3)	0.049 (2)	0.010 (2)	−0.0076 (19)	0.007 (2)
C15	0.070 (3)	0.069 (4)	0.058 (3)	0.027 (3)	−0.006 (2)	0.012 (3)
C16	0.094 (4)	0.047 (4)	0.078 (3)	0.028 (3)	0.015 (3)	0.021 (3)
C17	0.076 (3)	0.029 (2)	0.085 (3)	0.000 (2)	0.011 (3)	0.000 (2)
C18	0.051 (2)	0.037 (3)	0.065 (3)	−0.001 (2)	−0.0052 (19)	−0.001 (2)
C19	0.060 (3)	0.049 (3)	0.067 (3)	−0.008 (2)	−0.017 (2)	0.001 (2)
C20	0.063 (3)	0.058 (4)	0.074 (3)	0.005 (3)	−0.011 (2)	0.018 (3)
C21	0.042 (2)	0.099 (5)	0.056 (2)	0.012 (3)	−0.0130 (17)	−0.011 (3)
C22	0.061 (3)	0.091 (5)	0.089 (4)	−0.002 (3)	−0.021 (3)	−0.036 (4)
C23	0.066 (3)	0.049 (3)	0.084 (3)	0.009 (3)	−0.018 (3)	−0.012 (3)

### Geometric parameters (Å, °)

**Table:**

Fe1—O2	1.897 (2)	C8—C13	1.417 (6)
Fe1—O1	2.105 (2)	C8—C9	1.433 (5)
Fe1—N1	2.106 (3)	C9—C10	1.409 (5)
Fe1—N2	2.254 (3)	C10—C11	1.364 (6)
Fe1—Cl1	2.2938 (10)	C10—H10	0.9300
Fe1—Cl2	2.3709 (12)	C11—C12	1.403 (7)
N1—C3	1.280 (4)	C11—H11	0.9300
N1—C2	1.462 (4)	C12—C13	1.344 (7)
N2—C18	1.331 (6)	C12—H12	0.9300
N2—C14	1.336 (5)	C13—H13	0.9300
N3—C19	1.312 (7)	C14—C15	1.375 (7)
N3—C23	1.319 (6)	C14—H14	0.9300
O1—C1	1.439 (4)	C15—C16	1.379 (8)
O1—H1	0.8200	C15—H15	0.9300
O2—C5	1.313 (4)	C16—C17	1.357 (7)
C1—C2	1.513 (4)	C16—H16	0.9300
C1—H1A	0.9700	C17—C18	1.379 (6)
C1—H1B	0.9700	C17—H17	0.9300
C2—H2A	0.9700	C18—H18	0.9300
C2—H2B	0.9700	C19—C20	1.393 (7)
C3—C4	1.440 (5)	C19—H19	0.9300
C3—H3	0.9300	C20—C21	1.344 (9)
C4—C5	1.414 (5)	C20—H20	0.9300
C4—C9	1.455 (5)	C21—C22	1.345 (9)
C5—C6	1.424 (5)	C21—H21	0.9300
C6—C7	1.348 (6)	C22—C23	1.361 (8)
C6—H6	0.9300	C22—H22	0.9300
C7—C8	1.402 (6)	C23—H23	0.9300
C7—H7	0.9300		
			
O2—Fe1—O1	163.37 (10)	C6—C7—H7	119.0
O2—Fe1—N1	86.33 (10)	C8—C7—H7	119.0
O1—Fe1—N1	77.32 (9)	C7—C8—C13	122.2 (4)
O2—Fe1—N2	91.01 (11)	C7—C8—C9	119.5 (3)
O1—Fe1—N2	84.14 (11)	C13—C8—C9	118.3 (4)
N1—Fe1—N2	83.39 (11)	C10—C9—C8	117.8 (3)
O2—Fe1—Cl1	102.90 (8)	C10—C9—C4	123.7 (3)
O1—Fe1—Cl1	92.81 (7)	C8—C9—C4	118.5 (3)
N1—Fe1—Cl1	167.24 (9)	C11—C10—C9	121.6 (4)
N2—Fe1—Cl1	87.61 (8)	C11—C10—H10	119.2
O2—Fe1—Cl2	94.40 (9)	C9—C10—H10	119.2
O1—Fe1—Cl2	88.73 (8)	C10—C11—C12	120.3 (4)
N1—Fe1—Cl2	90.00 (9)	C10—C11—H11	119.9
N2—Fe1—Cl2	171.18 (9)	C12—C11—H11	119.9
Cl1—Fe1—Cl2	97.95 (4)	C13—C12—C11	119.9 (4)
C3—N1—C2	119.9 (3)	C13—C12—H12	120.0
C3—N1—Fe1	126.5 (2)	C11—C12—H12	120.0
C2—N1—Fe1	113.6 (2)	C12—C13—C8	121.9 (4)
C18—N2—C14	117.8 (4)	C12—C13—H13	119.0
C18—N2—Fe1	121.5 (3)	C8—C13—H13	119.0
C14—N2—Fe1	120.7 (3)	N2—C14—C15	122.2 (5)
C19—N3—C23	117.5 (3)	N2—C14—H14	118.9
C1—O1—Fe1	114.1 (2)	C15—C14—H14	118.9
C1—O1—H1	109.5	C14—C15—C16	119.4 (4)
Fe1—O1—H1	107.4	C14—C15—H15	120.3
C5—O2—Fe1	131.7 (2)	C16—C15—H15	120.3
O1—C1—C2	106.1 (3)	C17—C16—C15	118.5 (5)
O1—C1—H1A	110.5	C17—C16—H16	120.7
C2—C1—H1A	110.5	C15—C16—H16	120.7
O1—C1—H1B	110.5	C16—C17—C18	119.2 (5)
C2—C1—H1B	110.5	C16—C17—H17	120.4
H1A—C1—H1B	108.7	C18—C17—H17	120.4
N1—C2—C1	108.3 (3)	N2—C18—C17	122.9 (4)
N1—C2—H2A	110.0	N2—C18—H18	118.6
C1—C2—H2A	110.0	C17—C18—H18	118.6
N1—C2—H2B	110.0	N3—C19—C20	122.7 (5)
C1—C2—H2B	110.0	N3—C19—H19	118.7
H2A—C2—H2B	108.4	C20—C19—H19	118.7
N1—C3—C4	125.4 (3)	C21—C20—C19	118.3 (5)
N1—C3—H3	117.3	C21—C20—H20	120.8
C4—C3—H3	117.3	C19—C20—H20	120.8
C5—C4—C3	121.8 (3)	C20—C21—C22	119.1 (4)
C5—C4—C9	119.1 (3)	C20—C21—H21	120.5
C3—C4—C9	119.1 (3)	C22—C21—H21	120.5
O2—C5—C4	123.2 (3)	C21—C22—C23	119.8 (6)
O2—C5—C6	117.2 (3)	C21—C22—H22	120.1
C4—C5—C6	119.6 (3)	C23—C22—H22	120.1
C7—C6—C5	121.1 (4)	N3—C23—C22	122.6 (5)
C7—C6—H6	119.5	N3—C23—H23	118.7
C5—C6—H6	119.5	C22—C23—H23	118.7
C6—C7—C8	122.1 (4)		
			
O2—Fe1—N1—C3	12.9 (3)	C3—C4—C5—O2	−4.1 (5)
O1—Fe1—N1—C3	−170.2 (3)	C9—C4—C5—O2	176.0 (3)
N2—Fe1—N1—C3	104.4 (3)	C3—C4—C5—C6	177.2 (3)
Cl1—Fe1—N1—C3	149.8 (3)	C9—C4—C5—C6	−2.8 (5)
Cl2—Fe1—N1—C3	−81.5 (3)	O2—C5—C6—C7	−179.5 (4)
O2—Fe1—N1—C2	−167.0 (3)	C4—C5—C6—C7	−0.7 (6)
O1—Fe1—N1—C2	9.8 (2)	C5—C6—C7—C8	1.7 (7)
N2—Fe1—N1—C2	−75.6 (3)	C6—C7—C8—C13	−178.5 (4)
Cl1—Fe1—N1—C2	−30.2 (5)	C6—C7—C8—C9	0.8 (7)
Cl2—Fe1—N1—C2	98.5 (3)	C7—C8—C9—C10	177.3 (4)
O2—Fe1—N2—C18	−146.3 (3)	C13—C8—C9—C10	−3.4 (5)
O1—Fe1—N2—C18	49.6 (3)	C7—C8—C9—C4	−4.2 (5)
N1—Fe1—N2—C18	127.5 (3)	C13—C8—C9—C4	175.1 (4)
Cl1—Fe1—N2—C18	−43.5 (3)	C5—C4—C9—C10	−176.5 (3)
O2—Fe1—N2—C14	34.9 (3)	C3—C4—C9—C10	3.6 (5)
O1—Fe1—N2—C14	−129.1 (3)	C5—C4—C9—C8	5.1 (5)
N1—Fe1—N2—C14	−51.3 (3)	C3—C4—C9—C8	−174.8 (3)
Cl1—Fe1—N2—C14	137.8 (3)	C8—C9—C10—C11	3.8 (6)
O2—Fe1—O1—C1	29.9 (5)	C4—C9—C10—C11	−174.6 (4)
N1—Fe1—O1—C1	19.0 (3)	C9—C10—C11—C12	−0.6 (7)
N2—Fe1—O1—C1	103.5 (3)	C10—C11—C12—C13	−3.2 (8)
Cl1—Fe1—O1—C1	−169.2 (3)	C11—C12—C13—C8	3.5 (8)
Cl2—Fe1—O1—C1	−71.3 (3)	C7—C8—C13—C12	179.1 (5)
O1—Fe1—O2—C5	−36.4 (6)	C9—C8—C13—C12	−0.2 (7)
N1—Fe1—O2—C5	−25.7 (3)	C18—N2—C14—C15	1.4 (6)
N2—Fe1—O2—C5	−109.0 (3)	Fe1—N2—C14—C15	−179.8 (3)
Cl1—Fe1—O2—C5	163.2 (3)	N2—C14—C15—C16	−0.3 (7)
Cl2—Fe1—O2—C5	64.0 (3)	C14—C15—C16—C17	−1.3 (8)
Fe1—O1—C1—C2	−42.2 (4)	C15—C16—C17—C18	1.8 (8)
C3—N1—C2—C1	145.1 (4)	C14—N2—C18—C17	−0.9 (6)
Fe1—N1—C2—C1	−34.9 (4)	Fe1—N2—C18—C17	−179.7 (3)
O1—C1—C2—N1	48.7 (5)	C16—C17—C18—N2	−0.7 (7)
C2—N1—C3—C4	179.1 (4)	C23—N3—C19—C20	0.5 (7)
Fe1—N1—C3—C4	−0.9 (5)	N3—C19—C20—C21	−0.3 (8)
N1—C3—C4—C5	−7.3 (6)	C19—C20—C21—C22	−0.8 (7)
N1—C3—C4—C9	172.6 (4)	C20—C21—C22—C23	1.7 (8)
Fe1—O2—C5—C4	25.9 (5)	C19—N3—C23—C22	0.3 (8)
Fe1—O2—C5—C6	−155.4 (3)	C21—C22—C23—N3	−1.5 (10)

### Hydrogen-bond geometry (Å, °)

**Table:**

Cg is the centroid of the C4–C9 ring.

**Table:**

*D*—H···*A*	*D*—H	H···*A*	*D*···*A*	*D*—H···*A*
O1—H1···N3	0.82	1.81	2.617 (4)	168
C12—H12···Cg^i^	0.93	2.71	3.594 (5)	159
C16—H16···Cg^ii^	0.93	2.78	3.653 (6)	157

Symmetry codes: (i) −*x*, *y*−1/2, −*z*; (ii) *x*, *y*+1, *z*.
